# Osteopathic Treatment of Postprandial Cardiovascular Symptoms Suggestive of Altered Autonomic Regulation: A Case Report

**DOI:** 10.7759/cureus.108430

**Published:** 2026-05-07

**Authors:** Harbi Shehadeh

**Affiliations:** 1 Osteopathy, School of Osteopathy of Madrid, Madrid, ESP; 2 Osteopathy, Private Practice in Physiotherapy and Osteopathy, Madrid, ESP

**Keywords:** autonomic modulation, heart rate variability, osteopathic manipulative treatment, postprandial hypotension, vagus nerve

## Abstract

Postprandial cardiovascular symptoms may be associated with alterations in autonomic regulation, particularly when the postprandial decrease in blood pressure is not accompanied by an adequate compensatory increase in heart rate. We report the case of an 84-year-old man with a long-standing history of postprandial symptoms, characterised by dizziness, generalised weakness, fatigue, and presyncope occurring between 10 and 30 minutes after food intake, with improvement in the supine position. The patient presented with postprandial hypotension without an appropriate increase in heart rate. A structured osteopathic manipulative treatment (OMT) protocol was applied over four weeks, targeting regions associated with vagal pathways. Symptom severity was assessed using the Orthostatic Hypotension Symptom Assessment (OHSA) subscale, and haemodynamic parameters were recorded both in the clinical setting and at home, alongside evaluation of autonomic function through heart rate variability (HRV). Following the intervention, the mean OHSA score decreased from 2.67 to 1.33 and remained stable at follow-up. Home monitoring showed a reduction in the magnitude of the postprandial blood pressure drop during intermediate periods, accompanied by a consistent increase in heart rate after food intake. In the clinical setting, consistent reductions in both blood pressure and heart rate were observed. HRV analysis revealed a decrease in low-frequency (LF) power and in LF in normalised units (LFnu), alongside a progressive increase in high-frequency (HF) power and in HF in normalised units (HFnu), with a reduction in the LF/HF ratio. Overall, these findings suggest a possible association between OMT and clinical improvement, accompanied by haemodynamic and autonomic changes consistent with autonomic modulation, potentially at the vagal level. However, these results should be interpreted with caution due to the single-case nature of the study, and further research with larger sample sizes and controlled designs is required to confirm these findings.

## Introduction

The vagus nerve constitutes a key component of the autonomic nervous system and functions as a mixed nerve, comprising approximately 80% afferent fibres and 20% efferent fibres, primarily innervating visceral organs. Through these connections, it is involved in the regulation of visceral functions, including gastrointestinal motility and heart rate control. It also contributes to bidirectional interactions between the central nervous system and the gastrointestinal tract, forming part of the gut-brain axis. Vagal afferents respond to mechanical, chemical, and hormonal stimuli originating from the gastrointestinal tract, which are centrally integrated to generate autonomic responses that modulate digestive functions such as motility [1].

From a neurophysiological perspective, the regulation of vagal activity is based on reflex and integrative circuits within the brainstem, particularly the dorsal vagal complex, which includes the nucleus tractus solitarius, the dorsal motor nucleus of the vagus, and the area postrema. These structures receive visceral afferent input from thoracic and abdominal organs and, following central processing, modulate parasympathetic efferent output to target organs. In the gastrointestinal context, this control is primarily mediated through vagovagal reflexes, which regulate motility and digestive secretion in response to food intake, playing a fundamental role in autonomic regulation and physiological homeostasis [2].

Food intake induces an adaptive autonomic response characterised by changes in the sympathovagal balance and postprandial haemodynamic changes that promote the redistribution of blood flow towards the splanchnic circulation [3]. This response involves a complex interaction between reduced systemic vascular resistance due to splanchnic vasodilation and compensatory autonomic mechanisms, including increases in heart rate and vascular tone, which are necessary to maintain haemodynamic stability after food intake. Vagal tone can be assessed non-invasively through heart rate variability (HRV) analysis, which is considered an indirect marker of sympathovagal balance [1]. Spectral analysis of HRV has demonstrated a reduction in cardiac vagal modulation following food intake [3]. Similarly, studies assessing cardiac vagal tone have reported a significant decrease in postprandial vagal activity, confirming the involvement of the autonomic nervous system in the cardiovascular response to feeding [4].

When these mechanisms of autonomic regulation are disrupted, clinical manifestations resulting from autonomic imbalance may occur. In this context, postprandial hypotension is characterised as a condition associated with dysfunction of autonomic compensatory mechanisms, characterised by a decrease in blood pressure following food intake. Evidence suggests that this phenomenon is not primarily due to increased splanchnic pooling, but rather to inadequate cardiovascular compensation for the physiological reduction in blood pressure induced by feeding, which under normal conditions is counteracted by increases in peripheral vascular resistance, heart rate, and cardiac output. These alterations reflect impaired cardiovascular regulation associated with an insufficient postprandial sympathetic response, including attenuated sympathetic activation and an inadequate chronotropic response [5]. However, the clinical characterisation of patients with reproducible postprandial symptoms associated with alterations in vagal modulation remains limited, particularly in cases of early onset and in the absence of surgical history or structural pathology.

Within the context of autonomic regulation, the osteopathic approach has been proposed as a potential intervention capable of influencing autonomic nervous system function. Studies in healthy subjects have shown that osteopathic manipulative treatment (OMT) can induce acute changes in cardiovascular autonomic activity, as evidenced by alterations in HRV parameters, including increases in high-frequency (HF) power, in some cases decreases in low-frequency (LF) power, and reductions in the LF/HF ratio, as well as changes in parameters expressed in normalised units (LFnu and HFnu). These findings are consistent with increased parasympathetic modulation compared with control conditions [6-8], as well as increases in time-domain indices such as the standard deviation of normal-to-normal intervals (SDNN) [8]. However, these parameters represent indirect measures of autonomic activity, and their interpretation as indicators of sympathovagal balance remains controversial. Furthermore, the available evidence is primarily derived from experimental studies conducted under baseline conditions, which limits its applicability to clinical contexts characterised by dynamic alterations in autonomic regulation, such as the postprandial period.

The aim of this case report is to describe the clinical course of a patient with postprandial autonomic cardiovascular symptoms following osteopathic intervention and their potential relationship with alterations in autonomic regulation. Given the role of vagal pathways in cardiovascular and gastrointestinal modulation, as well as their involvement in the integration of postprandial visceral responses, it is proposed that altered vagal modulation may play a central role in the observed symptomatology, in interaction with other mechanisms of the autonomic nervous system, including the sympathetic response and baroreflex mechanisms.

## Case presentation

An 84-year-old man presented with recurrent episodes of postprandial discomfort following his largest meal of the day since 2003, when he was clinically diagnosed with a syndrome consistent with dumping in the absence of prior gastric surgery. Postprandial symptoms progressively increased in both frequency and intensity, later extending to breakfast and dinner. In July 2021, a gastric emptying study demonstrated an accelerated pattern consistent with dumping. In November 2021, during cardiology evaluation, the possible involvement of altered postprandial autonomic modulation with vagal predominance was considered. Repeated measurements showed low blood pressure values, approximately 100/60 mmHg. Management has been based on dietary measures aimed at controlling symptoms. Episodes typically occur between 10 and 30 minutes after food intake and include dizziness, generalised weakness, fatigue, and a sensation of lightheadedness, occasionally accompanied by cold sweats and presyncopal symptoms. Symptoms improve in the supine position, usually resolving after approximately 30 minutes of rest.

Relevant medical history includes cholecystectomy (1969), right hip arthroplasty (2007), infrarenal abdominal aortic aneurysm and right common iliac artery aneurysm (2014), and left inguinal hernioplasty (2025). Given that dumping syndrome is typically associated with gastric surgery, the only potentially related procedure would be the cholecystectomy; however, the long interval between this intervention and the onset of symptoms makes a causal relationship unlikely. His regular medication included acetylsalicylic acid and simvastatin.

Osteopathic assessment revealed somatic dysfunctions characterised by restricted mobility in the craniocervical region, as well as tissue restrictions in the suboccipital region, anterior cervical region, and anterior mediastinum. The clinical pattern and the suspicion of autonomic dysfunction suggested that vagal modulation could play a relevant role in the pathophysiology of the condition, guiding the subsequent clinical approach.

Symptom assessment

The severity of orthostatic symptoms was assessed using the Orthostatic Hypotension Questionnaire (OHQ), an instrument designed to quantify symptom burden and the functional impact of neurogenic orthostatic hypotension (NOH). It comprises two subscales: the Orthostatic Hypotension Symptom Assessment (OHSA) and the Orthostatic Hypotension Daily Activity Scale (OHDAS). In its validation study, the OHQ demonstrated adequate construct validity [9]. As the aim was to evaluate symptom severity exclusively, only the OHSA subscale was used, as it specifically assesses symptom severity and has demonstrated adequate psychometric properties as a subscale of the OHQ, including internal consistency. It includes six items (dizziness, lightheadedness, feeling faint, or feeling like you might black out; problems with vision; weakness; fatigue; trouble concentrating; and head or neck discomfort), each scored from 0 (no symptoms) to 10 (worst imaginable severity) based on the average severity over the previous seven days. The final score was calculated as the arithmetic mean of the items, with a value of 0 assigned to symptoms not experienced.

In accordance with the OHQ instructions, the patient was instructed to rate only symptoms associated with standing that improved in the supine position in order to isolate symptoms dependent on postural haemodynamic changes. Although the OHSA was developed for NOH, its design is based on the assessment of a clinical pattern characterised by orthostatic dependence and transient cerebral hypoperfusion. In the present case, episodes were triggered in the postprandial period and were included in the assessment only when they exhibited orthostatic dependence. This partial overlap justifies its use as a standardised measure of symptom severity in this clinical context. Nevertheless, its use was considered exploratory and not intended for diagnostic purposes, and it was employed as a tool for longitudinal assessment, with cautious interpretation of the results. The questionnaire was administered prior to each of the four sessions. The assessment conducted prior to session 1 was used as the reference value. An additional follow-up assessment was performed one week after the final session by telephone. In total, five OHSA assessments were obtained during the follow-up period.

Haemodynamic assessment

Blood pressure was measured using an automated oscillometric upper-arm device (Omron M2, Omron Healthcare Co., Ltd., Kyoto, Japan), validated according to the International Protocol of the European Society of Hypertension (ESH) [10]. Measurements were performed in accordance with ESH recommendations for clinical blood pressure measurement and home blood pressure monitoring (HBPM) [11], with heart rate recorded simultaneously under standardised conditions. All measurements were performed in the seated position on the left arm to ensure consistency.

In the clinical setting, measurements were obtained during four sessions at weekly intervals. At each session, measurements were taken before and after the therapeutic intervention, following a seated rest period of at least 5 minutes. For each time point, three consecutive measurements were obtained at 1-minute intervals, and the mean of the last two was used for analysis.

In addition, a structured HBPM protocol was implemented. The patient was instructed in the correct use of the device, and the same device was used both in the clinic and at home to ensure measurement consistency. A one-week reference period was recorded prior to the start of treatment. Subsequently, four consecutive weekly monitoring periods were conducted in association with each therapeutic session, including the period following the final intervention, resulting in a total of five weekly periods. During each period, the patient performed daily measurements at two time points: before the main meal, after at least 5 minutes of seated rest, and 10 minutes after its completion, maintaining standardised postural conditions. On each occasion, three consecutive measurements were obtained at 1-minute intervals, and the mean of the last two was used. For analysis, weekly mean values of blood pressure and heart rate were calculated separately for preprandial and postprandial conditions in each period in order to obtain representative estimates of haemodynamic parameters.

Autonomic function assessment

Autonomic function was assessed using HRV recordings, following established methodological standards for short-term recordings [12]. In accordance with this framework, measurements were performed after a resting period of at least 5 minutes, followed by a 5-minute HRV recording, conducted immediately before each session. The same procedure was repeated after each session. Assessments were carried out in the supine position, in a quiet environment with dim lighting, with the legs uncrossed and avoiding speaking or movement during the recording. The patient was instructed to breathe spontaneously and remain relaxed throughout the procedure. The patient underwent a total of four sessions at weekly intervals, with recordings performed before and after each session.

**Figure 1:**
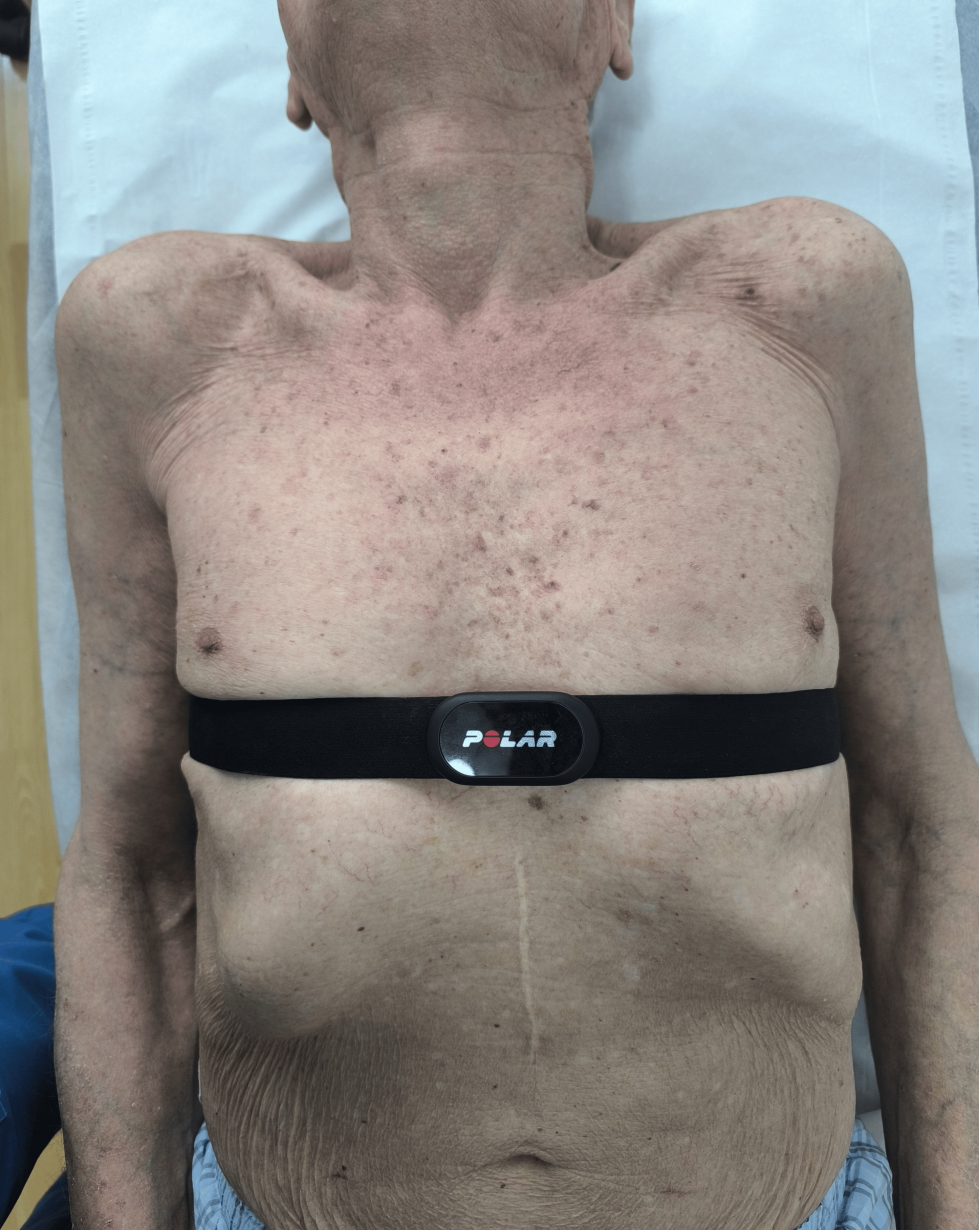
Polar H10 chest strap (Polar Electro Oy, Kempele, Finland) used for heart rate variability recording.

Cardiac signal was recorded using a Polar H10 chest strap (Polar Electro Oy, Kempele, Finland) (Figure 1), synchronised via Bluetooth with the Kubios HRV Mobile application (version 1.6.6; Kubios Oy, Kuopio, Finland). R-R interval processing included artefact correction using the automatic processing implemented within the application, applying the standard settings. HRV parameters were calculated automatically within the application, without additional processing. The Polar H10 sensor has demonstrated high agreement in R-R interval detection [13]. This methodological approach, based on the use of validated wearable devices and dedicated software for HRV analysis in short-term recordings under controlled conditions, has been previously described in the recent literature [14]. Time-domain parameters were analysed, specifically the root mean square of successive differences (RMSSD) and the SDNN, as well as frequency-domain parameters, expressed in normalised units (LFnu and HFnu); LF power (0.04-0.15 Hz); HF power (0.15-0.40 Hz), both expressed in absolute values (ms²); and the LF/HF ratio.

Osteopathic intervention

The treatment consisted of the application of an OMT approach using gentle manual techniques. It was delivered over four weekly sessions, with an approximate duration of 20 minutes per session (2-3 minutes per technique). During all sessions, the patient remained in the supine position. The techniques were applied following a defined order, which enhances their reproducibility in clinical practice. The therapeutic approach was established based on the anatomical course of the vagus nerve [15] and was applied to regions related to its trajectory, in accordance with routine osteopathic clinical practice.

The following techniques were applied: jugular foramen technique in the occipitomastoid region. Suboccipital inhibition is a soft tissue technique involving sustained pressure on the suboccipital musculature. Suboccipital decompression in the occipito-atlantal region through contact with the occipital bone and sustained traction. Cervical myofascial release was applied to cervical tissues in the region of the carotid sheath. The anterior mediastinum technique was applied to the anterior thoracic tissues. Treatment of the upper abdominal viscera, including structures such as the stomach and the hepatoduodenal area. Finally, the CV4 technique (compression of the fourth ventricle), a cranial technique involving sustained compression of the occipital squama, was incorporated at the end of the protocol. All techniques were applied using standardised manual contacts and gentle, sustained pressures in accordance with commonly described osteopathic procedures. During the follow-up period, the patient maintained his usual pharmacological treatment unchanged and did not receive additional interventions aimed at modulating autonomic function. No adverse effects or undesired events were reported during the intervention period.

Results

The mean OHSA score showed a decrease across study time points, from 2.67 prior to session 1 to 1.33 prior to session 4, and remained stable at the one-week follow-up. The reduction was observed mainly in the weakness and fatigue items, which decreased from 6 to 3 and from 6 to 2, respectively. Dizziness/lightheadedness decreased from 4 to 3 and subsequently remained stable. The items related to problems with vision, trouble concentrating, and head or neck discomfort remained at 0 throughout the study period (Table 1).

**Table 1:** Orthostatic Hypotension Symptom Assessment (OHSA) scores at each study time point OHSA items are scored from 0 (no symptoms) to 10 (worst imaginable severity), reflecting the average severity over the previous seven days. The mean OHSA score represents the arithmetic mean of the six item scores. Assessments were performed prior to each session and at one-week follow-up. Item wording has been abbreviated for clarity.

Time point	Dizziness/lightheadedness	Problems with vision	Weakness	Fatigue	Trouble concentrating	Head/neck discomfort	Mean OHSA score
Prior to session 1	4	0	6	6	0	0	2.67
Prior to session 2	3	0	4	4	0	0	1.83
Prior to session 3	3	0	3	3	0	0	1.50
Prior to session 4	3	0	3	2	0	0	1.33
Follow-up	3	0	3	2	0	0	1.33

In the clinical setting, blood pressure and heart rate decreased after each session. When averaged across sessions, using values obtained before and after each session, systolic blood pressure (SBP) decreased from 101.9 to 96.3 mmHg (-5.6 mmHg), diastolic blood pressure (DBP) from 69.0 to 65.4 mmHg (-3.6 mmHg), and heart rate (HR) from 67.5 to 63.6 beats per minute (bpm; -3.9 bpm), showing a consistent pattern across the four sessions (Table 2).

**Table 2:** Blood pressure and heart rate measured before and after each session Values represent the mean of the last two consecutive measurements obtained in the clinical setting before and after each session. For reference, normal resting blood pressure in adults is typically around 120/80 mmHg, and normal resting heart rate ranges between 60 and 100 bpm. SBP: systolic blood pressure; DBP: diastolic blood pressure; HR: heart rate; Pre/Post: before and after the session; bpm: beats per minute.

Session	SBP Pre (mmHg)	SBP Post (mmHg)	DBP Pre (mmHg)	DBP Post (mmHg)	HR Pre (bpm)	HR Post (bpm)
1	105.0	95.0	71.0	64.0	66.0	64.0
2	102.5	97.0	67.5	66.5	68.5	63.0
3	100.0	95.0	71.0	67.0	67.0	64.0
4	100.0	98.0	66.5	64.0	68.5	63.5

For HBPM, five consecutive weekly periods were analysed, including one reference period, three intermediate periods, and one follow-up period. Preprandial values showed variability across the study periods, with higher values observed during the intermediate periods and a tendency towards stabilisation in the final period. In all periods, postprandial values were lower than preprandial values. The reduction was more pronounced in the reference period, remained smaller during the intermediate periods, and increased again in the later periods. Heart rate showed a distinct pattern, with no postprandial increase in the initial period and an increase in the subsequent periods (Table 3).

**Table 3:** Weekly mean blood pressure and heart rate values under preprandial and postprandial conditions across study periods Values represent weekly means derived from daily home blood pressure measurements performed before and after the main meal. For reference, normal resting blood pressure in adults is typically around 120/80 mmHg, and normal resting heart rate ranges between 60 and 100 bpm. P1: week prior to treatment initiation (reference period); P2-P4: consecutive weeks between sessions; P5: week following the final session (follow-up); SBP: systolic blood pressure; DBP: diastolic blood pressure; HR: heart rate; Pre: preprandial; Post: postprandial; bpm: beats per minute.

Parameter	P1 Pre	P1 Post	P2 Pre	P2 Post	P3 Pre	P3 Post	P4 Pre	P4 Post	P5 Pre	P5 Post
SBP (mmHg)	108.3	93.6	107.6	100.6	119.9	110.7	115.8	103.3	117.9	103.1
DBP (mmHg)	74.7	59.6	74.8	69.9	82.2	75.1	77.3	70.0	81.1	71.4
HR (bpm)	73.9	72.6	70.9	77.4	70.4	76.9	71.1	76.0	71.1	77.4

**Figure 2:**
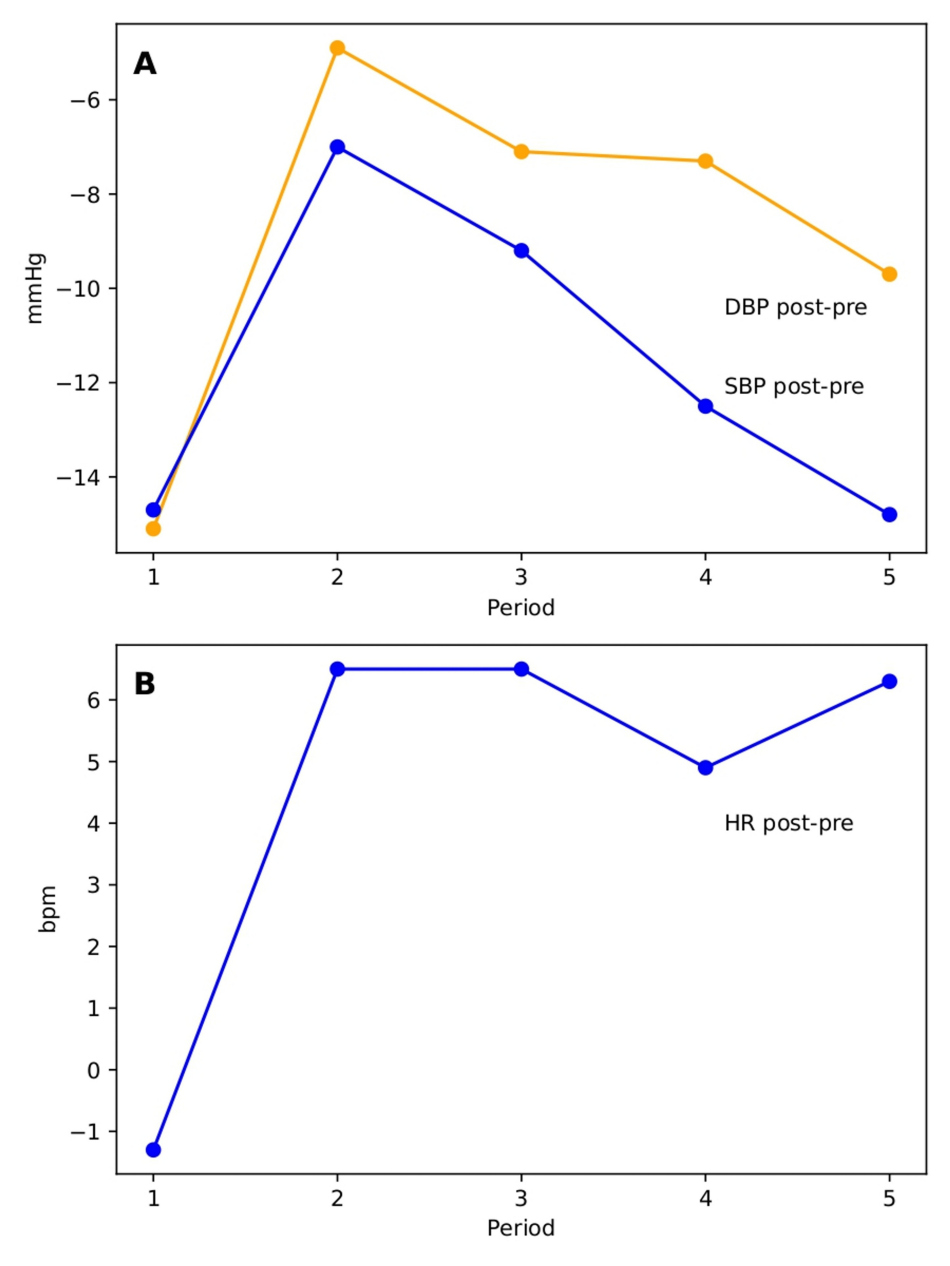
Postprandial differences in systolic blood pressure (SBP), diastolic blood pressure (DBP), and heart rate (HR) across study periods (A) Differences in SBP and DBP (mmHg). (B) Differences in HR (beats per minute, bpm). Values represent the difference between post and pre (post-pre) for each period. Negative values indicate a decrease after food intake, whereas positive values indicate an increase. Pre: preprandial; Post: postprandial

To characterise the postprandial haemodynamic response, the difference between postprandial and preprandial values was calculated for each period (Figure 2).

Regarding HRV parameters, global variability indices (RMSSD and SDNN) did not show relevant changes across sessions. LF power decreased after each session, whereas HF power tended to increase. A reduction in LFnu and an increase in HFnu were observed across sessions. Consequently, the LF/HF ratio showed a systematic decrease (Table 4).

**Table 4:** Heart rate variability (HRV) parameters before and after each session Values correspond to 5-minute HRV recordings obtained under resting conditions before and after each session. Frequency-domain parameters are expressed as absolute power (ms²) and in normalised units, representing relative proportions. RMSSD: root mean square of successive differences; SDNN: standard deviation of normal-to-normal intervals; LF: low frequency; HF: high frequency; LF/HF: low frequency to high frequency ratio; LFnu: low frequency in normalised units; HFnu: high frequency in normalised units.

Session	Time Point	RMSSD (ms)	SDNN (ms)	LF Power (ms²)	HF Power (ms²)	LFnu	HFnu	LF/HF Ratio
1	Pre-session	7	5.89	13.71	11.04	55.38	44.58	1.24
1	Post-session	7	5.30	7.12	13.22	34.99	64.94	0.54
2	Pre-session	7	5.87	11.86	13.78	46.22	53.70	0.86
2	Post-session	7	5.12	5.29	15.32	25.66	74.31	0.35
3	Pre-session	7	5.25	7.03	14.96	31.93	68.00	0.47
3	Post-session	7	5.55	6.09	15.62	28.02	71.92	0.39
4	Pre-session	7	5.09	7.52	11.38	39.74	60.17	0.66
4	Post-session	8	6.29	7.20	15.86	31.18	68.63	0.45

## Discussion

The vagus nerve plays a central role in the regulation of gastrointestinal motility, visceral sensitivity, and autonomic function. Reduced vagal tone has been associated with autonomic dysfunction, and its modulation has been proposed as a therapeutic target in various gastrointestinal disorders, particularly in the context of the gut-brain axis [1]. From a physiological perspective, the clinical improvement observed in this case may reflect, at least in part, modulation of sympathovagal balance, possibly mediated by vagovagal reflex mechanisms. In this regard, visceral vagal afferent innervation conveys information to the nucleus tractus solitarius, where it is integrated and generates parasympathetic efferent responses that modulate visceral function. Various visceral stimuli, including mechanical stimuli, may activate these afferents and trigger reflex responses at the level of the brainstem [2]. From this perspective, it is plausible to suggest that interventions targeting the upper abdominal viscera, such as those applied in the present case, may influence autonomic modulation through these circuits, although direct evidence in humans remains limited.

The observed reduction in the mean OHSA score (from 2.67 to 1.33) exceeds the minimal clinically important difference (MID, approximately 0.8-1.0 points) reported for this instrument, which is consistent with a clinically relevant symptomatic improvement. This change was primarily driven by reductions in weakness and fatigue, suggesting a clinically relevant improvement in the patient’s functional tolerance during the postprandial period. Although the MID was established at the population level, in this context, it is used in an indicative manner to estimate the magnitude of change observed in this patient. Nevertheless, these findings should be interpreted with caution, as the OHSA was developed and validated in patients with neurogenic orthostatic hypotension and not for the assessment of postprandial symptomatology. However, despite the predominantly postprandial trigger, the consistent association of symptoms with upright posture and their improvement in the supine position suggests a shared haemodynamic mechanism, based on episodes of transient cerebral hypoperfusion mediated by autonomic dysfunction. This supports the notion that the symptoms assessed are reasonably aligned with the clinical construct that the instrument is intended to measure.

Under physiological conditions, the postprandial decrease in blood pressure is typically compensated by an increase in peripheral vascular resistance, heart rate, and cardiac output, thereby maintaining haemodynamic stability following food intake [5]. During the initial home monitoring period, the patient exhibited a marked postprandial decline in blood pressure without a compensatory increase in heart rate, which may be consistent with an attenuated cardiovascular autonomic response. Following the intervention, a reduction in the postprandial blood pressure drop was observed during the intermediate periods, with a subsequent increase in the later period. In contrast, a consistent increase in heart rate after food intake was observed following the intervention, suggesting an improvement in the physiological compensatory response, particularly at the chronotropic level. In addition, in the clinical setting, a reduction in both blood pressure and heart rate was observed after the intervention. Taken together, these findings may be consistent with an improvement in haemodynamic adaptation, with particular relevance to the enhanced heart rate response to the postprandial haemodynamic challenge. The discrepancy observed between home and clinical measurements may reflect context-dependent variability, including potential influences of the clinical setting, such as the white coat effect, and natural physiological variability, despite the use of standardised measurement conditions.

HRV has been widely used as a non-invasive marker of vagal tone and sympathovagal balance, allowing indirect assessment of autonomic modulation. In the field of osteopathy, it represents a relevant tool for analysing the autonomic response to manual interventions, with changes in parasympathetic-related parameters having been reported. However, findings are inconsistent and do not allow firm conclusions to be drawn [16]. Overall, systematic reviews suggest that osteopathic interventions may induce changes in the autonomic nervous system, although the evidence is heterogeneous, of moderate methodological quality, and does not allow clear conclusions to be established regarding sympathetic or parasympathetic modulation [17].

In this context, HRV analysis provided additional insight into the autonomic response associated with the interventions. While global variability indices (RMSSD and SDNN) remained relatively stable, LF power decreased after each session, with more marked reductions during the initial sessions, whereas HF power increased progressively. Consistently, a reduction in LFnu and an increase in HFnu were observed. As a result, the LF/HF ratio decreased steadily from 1.24 in the first session to 0.45 in the final session. Overall, these changes in the spectral profile of HRV are consistent with modulation of autonomic control, suggesting a shift towards greater relative vagal modulation accompanied by a relative reduction in sympathetic influence. This pattern was observed consistently across sessions, suggesting an acute effect with a possible tendency towards progressive changes. Similar findings have been reported in experimental studies using the CV4 technique, in which a decrease in LF, an increase in HF, and a reduction in the LF/HF ratio were observed [18], as well as a comparable pattern in other studies involving healthy subjects [6-8].

The interpretation of the LF/HF ratio and frequency-domain parameters remains a matter of debate, particularly regarding their validity as indicators of sympathovagal balance, which has been widely questioned [19]. Therefore, these findings should be interpreted with caution. Given the single-case nature of the present study and the indirect nature of HRV-derived indices, which are influenced by multiple physiological factors, causal relationships cannot be established. In addition, potential contributions from non-specific effects, including contextual or placebo-related influences, as well as regression to the mean and natural physiological variability, cannot be excluded. Furthermore, the observed results may be influenced by the patient’s inherent physiological variability, which affects HRV as well as blood pressure, heart rate, and the postprandial haemodynamic responses.

Certain limitations should be considered when interpreting the present case. The postprandial symptomatology observed may be related to an impairment of cardiovascular compensatory mechanisms associated with ageing. In this regard, attenuation of the autonomic response, particularly reduced compensatory sympathetic activation and cardiovascular reflex mechanisms, has been described as one of the processes involved in the pathophysiology of postprandial hypotension in older individuals [5]. In addition, the patient had a previous diagnosis of dumping syndrome. Although this condition is typically associated with gastric or oesophageal surgery, cases without prior surgical history have also been reported, with the idiopathic form remaining controversial. From a pathophysiological perspective, dumping syndrome is characterised by the rapid transit of nutrients into the small intestine, which in its early phase may induce fluid shifts into the intestinal lumen and the release of gastrointestinal hormones, leading to vasomotor symptoms [20]. Therefore, it is not straightforward to determine whether the patient’s postprandial symptomatology is exclusively attributable to this mechanism, is related to an underlying autonomic dysfunction, or reflects a possible overlap of both processes.

Overall, the findings of the present case suggest that modulation of the autonomic nervous system may represent one of the mechanisms underlying the observed clinical improvement. This improvement likely reflects a complex interaction between haemodynamic and autonomic mechanisms, without it being possible to attribute it to a single predominant pathophysiological process. The results raise the possibility that certain manual interventions may influence autonomic regulation involved in digestive function, thereby reinforcing interest in exploring their role within the gut-brain axis.

Nevertheless, the single-case nature of the study and the absence of a control group limit the ability to establish a causal relationship between the osteopathic intervention and the observed clinical or autonomic changes. Future studies with larger sample sizes, controlled designs, and blinding procedures, incorporating objective measures such as HRV, will be required to more precisely evaluate the role of OMT in cardiovascular autonomic modulation in patients with postprandial symptomatology.

## Conclusions

This case report describes a favourable clinical course in a patient with postprandial cardiovascular autonomic symptoms following the application of an OMT protocol targeting regions associated with vagal pathways. The observed improvement was characterised not only by a reduction in symptom severity, but also by a more appropriate haemodynamic response to food intake, particularly reflected in the restoration of the postprandial heart rate increase. These findings were accompanied by changes in HRV parameters consistent with a shift towards greater autonomic modulation.

Although these results suggest a possible interaction between osteopathic intervention and autonomic regulation, causal relationships cannot be established. The complexity of the underlying pathophysiology, together with the single-case design, requires cautious interpretation. Nevertheless, this case highlights the potential relevance of autonomic modulation as a therapeutic target and supports further investigation of manual interventions in dynamic autonomic conditions such as the postprandial state. Future studies with larger sample sizes, controlled designs, and objective outcome measures are warranted to confirm these observations.

## References

[1] Bonaz B, Sinniger V, Pellissier S (2016). Vagal tone: effects on sensitivity, motility, and inflammation. Neurogastroenterol Motil.

[2] Browning KN, Travagli RA (2014). Central nervous system control of gastrointestinal motility and secretion and modulation of gastrointestinal functions. Compr Physiol.

[3] Lu CL, Zou X, Orr WC, Chen JD (1999). Postprandial changes of sympathovagal balance measured by heart rate variability. Dig Dis Sci.

[4] Kuo P, Bravi I, Marreddy U, Aziz Q, Sifrim D (2013). Postprandial cardiac vagal tone and transient lower esophageal sphincter relaxation (TLESR). Neurogastroenterol Motil.

[5] Luciano GL, Brennan MJ, Rothberg MB (2010). Postprandial hypotension. Am J Med.

[6] Henley CE, Ivins D, Mills M, Wen FK, Benjamin BA (2008). Osteopathic manipulative treatment and its relationship to autonomic nervous system activity as demonstrated by heart rate variability: a repeated measures study. Osteopath Med Prim Care.

[7] Ruffini N, D'Alessandro G, Mariani N, Pollastrelli A, Cardinali L, Cerritelli F (2015). Variations of high frequency parameter of heart rate variability following osteopathic manipulative treatment in healthy subjects compared to control group and sham therapy: randomized controlled trial. Front Neurosci.

[8] Giles PD, Hensel KL, Pacchia CF, Smith ML (2013). Suboccipital decompression enhances heart rate variability indices of cardiac control in healthy subjects. J Altern Complement Med.

[9] Kaufmann H, Malamut R, Norcliffe-Kaufmann L, Rosa K, Freeman R (2012). The Orthostatic Hypotension Questionnaire (OHQ): validation of a novel symptom assessment scale. Clin Auton Res.

[10] Topouchian J, Agnoletti D, Blacher J (2011). Validation of four automatic devices for self-measurement of blood pressure according to the international protocol of the European Society of Hypertension. Vasc Health Risk Manag.

[11] Stergiou GS, Palatini P, Parati G (2021). 2021 European Society of Hypertension practice guidelines for office and out-of-office blood pressure measurement. J Hypertens.

[12] (1996). Heart rate variability: standards of measurement, physiological interpretation and clinical use. Task Force of the European Society of Cardiology and the North American Society of Pacing and Electrophysiology. Circulation.

[13] Schaffarczyk M, Rogers B, Reer R, Gronwald T (2022). Validity of the Polar H10 sensor for heart rate variability analysis during resting state and incremental exercise in recreational men and women. Sensors (Basel).

[14] Hannon J, O'Hagan A, Lambe R, O'Grady B, Doherty C (2025). Associations between daily heart rate variability and self-reported wellness: a 14-day observational study in healthy adults. Sensors (Basel).

[15] Neuhuber WL, Berthoud HR (2021). Functional anatomy of the vagus system - emphasis on the somato-visceral interface. Auton Neurosci.

[16] Stępnik J, Czaprowski D, Kędra A (2024). Effect of manual osteopathic techniques on the autonomic nervous system, respiratory system function and head-cervical-shoulder complex-a systematic review. Front Med (Lausanne).

[17] Rechberger V, Biberschick M, Porthun J (2019). Effectiveness of an osteopathic treatment on the autonomic nervous system: a systematic review of the literature. Eur J Med Res.

[18] Curi AC, Maior Alves AS, Silva JG (2018). Cardiac autonomic response after cranial technique of the fourth ventricle (cv4) compression in systemic hypertensive subjects. J Bodyw Mov Ther.

[19] Billman GE (2013). The LF/HF ratio does not accurately measure cardiac sympatho-vagal balance. Front Physiol.

[20] Scarpellini E, Arts J, Karamanolis G (2020). International consensus on the diagnosis and management of dumping syndrome. Nat Rev Endocrinol.

